# Low-Cost Force Sensors Embedded in Physical Human–Machine Interfaces: Concept, Exemplary Realization on Upper-Body Exoskeleton, and Validation

**DOI:** 10.3390/s22020505

**Published:** 2022-01-10

**Authors:** Niclas Hoffmann, Samet Ersoysal, Gilbert Prokop, Matthias Hoefer, Robert Weidner

**Affiliations:** 1 Department of Production Technologies, Institute of Mechatronics, University of Innsbruck, 6020 Innsbruck, Austria; Niclas.Hoffmann@hsu-hh.de (N.H.); Samet.Ersoysal@uibk.ac.at (S.E.); Gilbert.Prokop@uibk.ac.at (G.P.); Matthias.Hoefer@uibk.ac.at (M.H.); 2 Laboratory of Manufacturing Technology, Helmut-Schmidt-University (University of the Federal Armed Forces Hamburg), 22043 Hamburg, Germany

**Keywords:** force sensor, pressure sensor, low cost, exoskeleton, wearable robot, human–machine interaction, interaction forces, evaluation, metrological comparison

## Abstract

In modern times, the collaboration between humans and machines increasingly rises, combining their respective benefits. The direct physical support causes interaction forces in human–machine interfaces, whereas their form determines both the effectiveness and comfort of the collaboration. However, their correct detection requires various sensor characteristics and remains challenging. Thus, this paper presents a developed low-cost sensor pad working with a silicone capsule and a piezoresistive pressure sensor. Its measurement accuracy is validated in both an isolated testing environment and a laboratory study with four test subjects (gender-balanced), and an application integrated in interfaces of an active upper-body exoskeleton. In the material-testing machine, it becomes apparent that the sensor pad generally features the capability of reliably determining normal forces on its surface until a certain threshold. This is also proven in the real application, where the measurement data of three sensor pads spatially embedded in the exoskeletal interface are compared to the data of an installed multi-axis load cell and a high-resolution flexible pressure map. Here, the consideration of three sensor pads potentially enables detection of exoskeletal support on the upper arm as well as “poor” fit conditions such as uneven pressure distributions that recommend immediate system adjustments for ergonomic improvements.

## 1. Introduction

In global economies and daily life situations, the demand for physical support in executing manual tasks continually rises to increase the productivity, ease the physical and cognitive workload, and improve the ergonomics and attractiveness of such workplaces. Here, from the connection between humans and support systems with their respective and unique properties can emerge an interacting, hybrid, and beneficial integral system [1]. For instance, humans feature distinct sensory abilities [2] and flexibility, whereas technical devices excel in terms of, e.g., repeat accuracy, endurance, or speed. Thus, and depending on the respective circumstances, multiple technical physical support systems such as collaborative robots, power tools, or exoskeletons are developed and generally accessible. These systems exhibit different technical functionalities and morphological structures [3].

Typically, any coupling between support system and user is made with physical interfaces, which generally evokes body-surface pressures [4] or body loads. Since both can be described as interaction forces, especially when the direction is crucial and the concrete contact surface area is undefined or unknown, this term is consistently used in the following. Their assessment might be of central interest for evaluating technical support systems in multiple dimensions. For instance, this could be the case for the risk assessment of collaborated robots in terms of ISO TS 15066 and the embeddedness of safety rules [5] as well as for ergonomic, customer-oriented, and targeted design decisions of, e.g., power tools based on analyzed grasp and clamping forces [6]. In addition, clinical applications are possible for observing, e.g., therapeutical compressions [7], rehabilitation motion trainings [8,9], or (tele-)medical diagnoses and treatments [10,11]. Tailored to the application example of exoskeletons as wearable, physical support systems with multiple possible characteristics in, e.g., power supply, interface design, or path of force [12], the assessment of the system’s effects on the users’ workload, kinematics, and wearing comfort is often of central interest [13]. In this case, evaluators can choose from a pool of applicable methods such as surveys, modeling, and simulation, or the analyses of muscular activities or movement patterns. However, the analyses of applied interaction forces in interfaces might enable a multidimensional evaluation with only one tool regarding the exoskeletal support (e.g., [14,15,16,17]), comfort (e.g., [14,18,19]), motion synchronicity (e.g., [15,20]), or movability (e.g., [21]). Additionally, and in case of being permanently embedded in the interface, the respective sensory data might be analyzed in real-time in order to detect poor system configurations and initiate system-sided adjustments [22]. However, support systems rarely feature the function of (permanently) detecting and analyzing occurring interaction forces thus far. In this case, the overall system integrability of the sensors is important and only inexpensive sensors should be applied in order to consider the budget and keep the system costs low. Furthermore, low-cost sensors enable designers or system developers to install more sensors for the realization of a higher area resolution or a broader sensing-coverage area.

The challenge principally remains in the accurate and comprehensive measurement of interaction forces—in particular in the low-budget area [4] and for detecting loads beyond normal forces. Pressure sensors applied on physical human–machine interfaces should feature linearity, low hysteresis, low drift, low temperature sensitivity, flexibility, thin construction, large measurement range, repeatability, ease of use, and cost-effectiveness [23,24]. Applicable measurement equipment, that proportionally maps external load to an electric quantity, ranges from, e.g., impedance, piezoelectric and magnetic are all principles for sensor measurements to barometric MEMS (Micro Electromechanical Systems) technology. Each technology features its own advantages and disadvantages. In accordance with multiple sensor requirements for wearable applications, piezoresistive sensors, capacitive sensors, or force-sensing resistors seem to be applicable. However, the performance of force-sensing resistors is known to be improvable in terms of linearity, stability, and repeatability [25,26,27,28]. Alternatively, high-resolution pressure sensors are a promising solution, measuring the compression of a medium in a flexible capsule and the resulting increase in the inner pressure caused by an external load on the capsule’s surface [24].

Taking this into consideration, Hoffmann et al. [29] have constructed a novel sensor pad with a piezoresistive pressure sensor inside of a flexible silicone capsule, whose basic construction principle is inspired by the previous work of Lee et al. [24]. The developed prototype has already exceled in an explorative laboratory study with five test scenarios by reliably detecting small interaction forces (between 0.2 newtons and 5.8 newtons) between a model of both an exoskeletal interface and human tissue as well as by exhibiting a confident performance in terms of stability, repeatability, accuracy, and sensitivity. Besides, the comparison of sensor data might even derive different states on the interface such as equal load distribution or occurring shear forces [29]. Consequently, this article expands the reliability check of the sensor pad and investigates the sensor pad’s behavior in both a standardized and isolated as well as a realistic testing environment. The key questions for the validation of the developed sensor pad refer to the mentioned requirements for pressure sensors applied to human–machine interfaces [23,24] as well as to insights from the previously applied explorative laboratory study [29]:

- Application in an isolated testing environment (experiment 1):
  ○ Concerning the measurement accuracy: Does the sensor pad provide a linear, stable, and repeatable determination of applied normal forces with low hysteresis in an expanded load range up to 200 newtons with a maximal duration of 10 min? The applied threshold of maximum 200 newtons is derived from the fact that commercially available cobots or supportive exoskeletons do not typically feature load eases beyond.
  ○ Concerning the ease of use: Is it necessary to fill the capsule with an incompressible medium such as water that influences the measured pressure in case of a spatial rotation of the sensor pad due to its varying hydrostatic head, and must be vented for preparing the sensor pad? Is the medium air a beneficial alternative?
  ○ Concerning the operational safety: Does the sensor pad and in particular the flexible silicone capsule withstand a mechanical load test increasing to 500 newtons? The applied threshold of maximum 500 newtons is derived from a measurement range of 200 kilopascals [23] that equals a load of 564 newtons considering the surface area of the sensor pad with 28.2 square centimeters.
- Application in a realistic testing environment with embedded sensor pads in the interface of an active shoulder exoskeleton (experiment 2):
  ○ Concerning the measurement accuracy: Does the sensor pad or its spatial array with three sensor pads provide reliable results for occurring interaction forces in human–machine interfaces?
  ○ Concerning the interpretability: Might the spatial array of the sensor pads embedded in the interface of an exoskeleton be capable of detecting “poor” load conditions such as uneven pressure distributions?

## 2. Materials and Methods

This section describes the construction and calibration of the sensor pad as well as its embedding in an exemplary exoskeletal interface. It also illustrates the test setups for the conducted experiments.

### 2.1. Basic Construction of the Sensor Pad and Its Embedding in an Exoskeletal Interface

**Figure 1:**
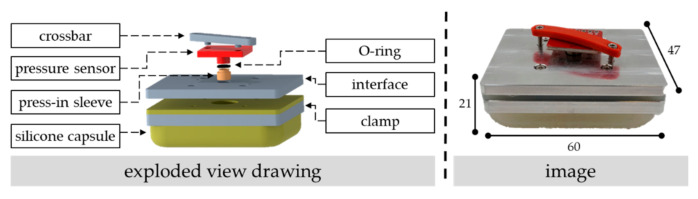
Exploded view drawing (**left**) and image (**right**) of the developed sensor pad. The dimensions are given in millimeters.

The construction of the sensor pad is detailly described in [29], and its basic construction is inspired by [24] using a “micro-sensor inside a flexible capsule filled with a pressure transmissible fluid”. As illustrated in Figure 1, it features a flexible silicon capsule cast from the shapeable, resilient, and skin-friendly multi-purpose silicone “Dragon Skin” with a shore hardness of 20A [30]. Its shape is formed by a milled mold, whereby vacuuming during the cast process releases air bubbles caused by previously mixing the two components of the silicone. The dimension of the silicone capsule is 60 mm × 47 mm × 10 mm with a wall thickness of 1 mm on the sides and 1.5 mm on the bottom and the top, respectively. The capsule is clamped between the interface and an aluminum plate with four mounting screws.

The sensor pad uses the piezoresistive pressure sensor MS5803-01BA that is placed on a breakout board for easy junction. A gel on the sensor’s surface protects the electronics against mediums such as liquids or air. In both experiments, the setup of the sensor is advantaged to a higher sampling rate. Thus, the selected oversampling ratio is 256 with a corresponding sensor resolution of 0.065 millibar and a response time of 0.5 milliseconds. The sensor has an accuracy of ±1.5 millibar and communicates via I^2^C with the microcontroller Arduino Uno. Further technical details of the sensor are documented in [31]. The selected sampling rate of the pressure sensor in the first and second experiment is set to 15 and 20 milliseconds, respectively. After the silicon capsule is filled with a medium (e.g., water or air), the pressure sensor is placed under the corresponding transfixion on the interface. The sealing is made with an O-ring placed between the outer diameter of the metal cap of the sensor and a pressed sleeve in the transfixion. Before the crossbar is clamped on the interface with two screws, the capsule is vented if the medium water is inside of the capsule. Tacks on the crossbar keep the sensor in the right position.

In addition, the low-cost characteristics and general availability of the sensor pad can be emphasized and ensured by the fact that the sensor pad is principally manufacturable with any economically accessible mold, clamp, and crossbar (e.g., milled or 3D-printed) as well as flexible silicon, a sensitive pressure sensor, and a programmable microcontroller. However, it needs to be mentioned that any modification in the sensor pads’ manufacturing might lead to changes in its later measurement performance.

**Figure 2:**
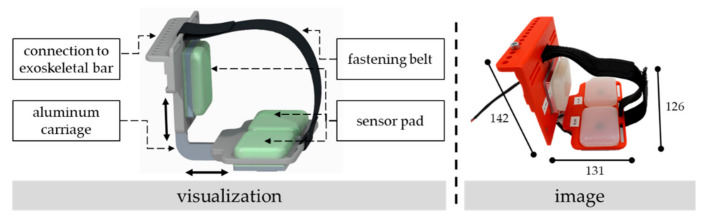
Visualization (**left**) and image (**right**) of the constructed exoskeletal interface with embedded force sensors and the ability to adjust the size with prepared holes in the aluminum carriage. The dimensions are given in millimeters.

To apply the sensor pad to an exoskeletal interface, multiple sensors are potentially needed to detect—apart from the supportive force—other load conditions on the interface caused by, e.g., changes in human soft tissues during motion or general mismatches in fitting [19,32]. In our case, the designed exoskeletal interface with embedded sensor pads takes up the spirit of the original interface of the used exoskeleton “Lucy 2.0” [33] consisting of a 3D-printed cup that is half-open, bolstered, clamped to the exoskeletal bar with two screws, and fixed to the upper arm with a fastening belt. Since the sensor pad provides both pressure detection and a bolstering of the interface, the sensor pad can be embedded in an interface without additional bolstering that might absorb forces and, thus, distort the measurements. As illustrated in Figure 2, the designed interface consists of one lateral module with one sensor for detecting forces to the upper arm mainly in the sagittal plane as well as one bottom module with two sensors for detecting forces and pressure distributions on the upper arm in the coronal plane. By sliding both modules on the connecting aluminum carriage and fixing them with screws in pre-defined holes, the size of the interface can be adjusted to different perimeters of the test subjects’ upper arm. A fastening belt fixes the interface when moving the arm.

### 2.2. Calibration of the Sensor Pad

The calibration process of the embedded pressure sensor is basically inspired by Wettenschwiler et al. [4] and mainly equal to the conducted sensor calibration in the previous, explorative study with the sensor pad [29]. After the barometric pressure is assessed under room temperature, every sensor pad is concentrically and consecutively loaded and unloaded with three different weights (100 g, 200 g, 500 g) that are placed on a 3D-printed sheet for equal pressure distribution on the silicon capsule’s surface. The resulting pressure increase after a load duration of five seconds is, respectively, documented. This procedure is repeated three times and all pressure increases for the same load against baseline are averaged. Based on this, a linear three-point calibration is made. The quality of this linear regression is sufficient, since R^2^ for all three pressure sensors is 0.999. It can be assumed that every calibrated sensor shows almost a linear behavior in the calibrated range. Based on the calibration, applied loads on the sensor can be measured as acting normal forces in newtons.

### 2.3. Test Setup—Material-Testing Machine (Experiment 1)

**Figure 3:**
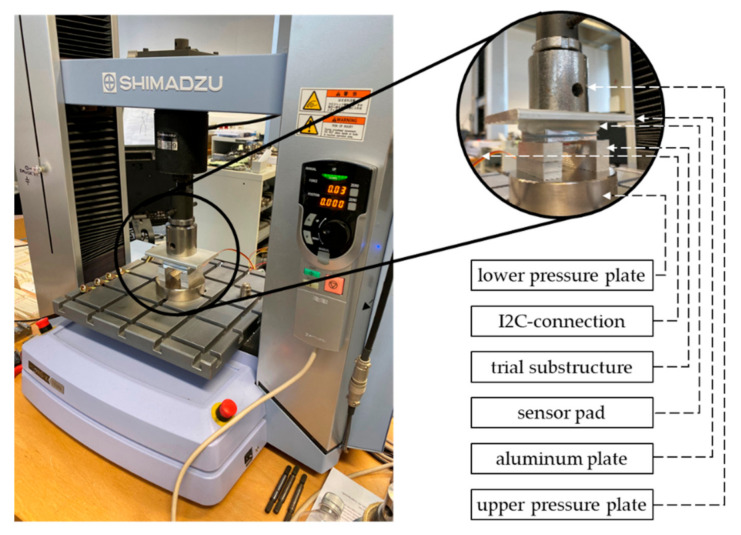
Clamp of the sensor pad in the material-testing machine.

To answer the introduced research questions according to the first experiment, the sensor pad is clamped in a precision universal testing machine by Shimadzu (AGX series) with a maximal load ability of 10.000 newtons (see Figure 3). Due to the external substructure of the pressure sensor, the sensor pad is placed upside down on a trial substructure with aluminum bars positioned on the lower pressure plate. Then, an aluminum plate (weight: 201.1 g) is inserted between the machine’s upper pressure plate so that the applied force is equally distributed on the surface of the flexible silicone capsule.

The following trials were conducted with the sensor pad clamped in the material testing machine:

- Trial 1: force ascent from 0 newtons to 500 newtons with an increase rate of 5 newtons per second, and force decrease to 0 newtons with a decrease rate of 5 newtons per second, one cycle, sensor pad filled with either water or air;
- Trial 2: force ascent from 0 newtons to 200 newtons with an increase rate of 5 newtons per second, hold for 600 s, and force decrease to 0 newtons with a decrease rate of 5 newtons per second, one cycle, sensor pad filled with air;
- Trial 3: force ascent from 0 newtons to 100 newtons with an increase rate of 5 newtons per second, hold for one second, and force decrease to 0 newtons with a decrease rate of 5 newtons per second, five cycles, sensor pad filled with air.

### 2.4. Test Setup—Exoskeleton Application (Experiment 2)

To assess the sensor pad’s performance in a real application, three sensor pads are embedded in the interface of the pneumatically actuated support system “Lucy 2.0” [33]. The exoskeleton is successively fixed to four test subjects (male: n = 2, female: n = 2; age: 25.0 ± 1.6 years, height: 171.8 ± 8.0 cm; weight: 69.8 ± 4.8 kg; upper-arm perimeter (arm stretched, measured in the middle of the upper arm): 27.5 ± 1.1 cm; upper-arm length (measured from shoulder acromion to olecranon): 34.5 ± 3.0 cm; shoulder width: 43.3 ± 6.2 cm) that performed a defined task at head level with the support system.

**Figure 4:**
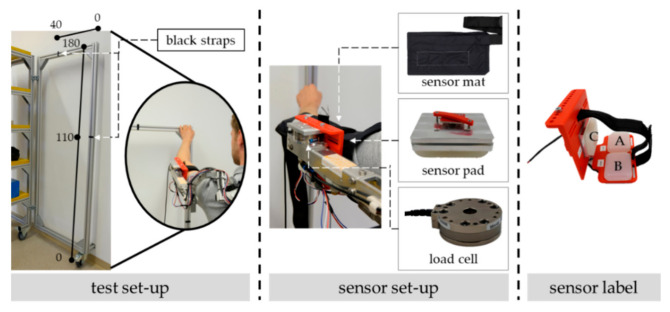
Illustration of the arranged comparing measurement equipment consisting of sensor mat and load cell as well as of the aluminum frame with black straps for marking motion endpoints. The dimensions are given in centimeters.

As illustrated in Figure 4 on the left, the left arm is repetitively moved on an aluminum frame between two marks with black straps. The operating range is vertical from 110 to 180 cm and horizontal from 0 to 40 cm.

The occurring interaction forces on the exoskeletal interface of the left arm are simultaneously detected by a pressure map and a load cell (see Figure 4 in the middle). Both methods are already applied on exoskeletal interfaces in other studies (see, e.g., [18,34], respectively). We used the thin, flexible, and high-resolution pressure map from XSensor Technology Corporation that is specifically designed for ergonomic assessments and ISO 17025 certified. Its sensing area is 12.7 cm × 25.4 cm with a resolution of 23.25 sensor points per square centimeter and a measurement range up to 10.34 newtons per square centimeter. In the experiment, the pressure map was wrinkle-freely placed between the sensor pad and the test subject’s skin so that the sensing area of the pressure map fully covered all three sensor pads. The fastening belt of the interface fixed the sensor map. Besides, we applied the force and torque sensor FTN-Mini-45 of Schunk with a maximum measurement range of ±290 newtons in F*_z_*, and ±145 newtons in F*_x_* and F*_y_* as well as ±5 newtons meter in M*_x_*, M*_y_*, and M*_z_*. Its resolution is 0.0625 newtons for F*_x_*, F*_y_*, and F*_z_*, whereas the resolution is 0.0005 newtons meter for M*_x_* and M*_y_* as well as 0.003 for M*_z_*. The sensor was placed between the aluminum frame of the exoskeleton and the physical interface so that it acted as a mounting for the physical interface. All measurements were executed in LabView to synchronize the different equipment data with the same timestamp.

In order to measure different load states and directions on the interface, certain combinations of sensor data need to be considered. Thus, and as illustrated in Figure 4 on the right, the sensor close to the elbow is labeled as A, close to the shoulder as B, and on the side as C.

## 3. Results

The results of the two conducted experiments are presented in the following.

### 3.1. Material-Testing Machine (Experiment 1)

**Figure 5:**
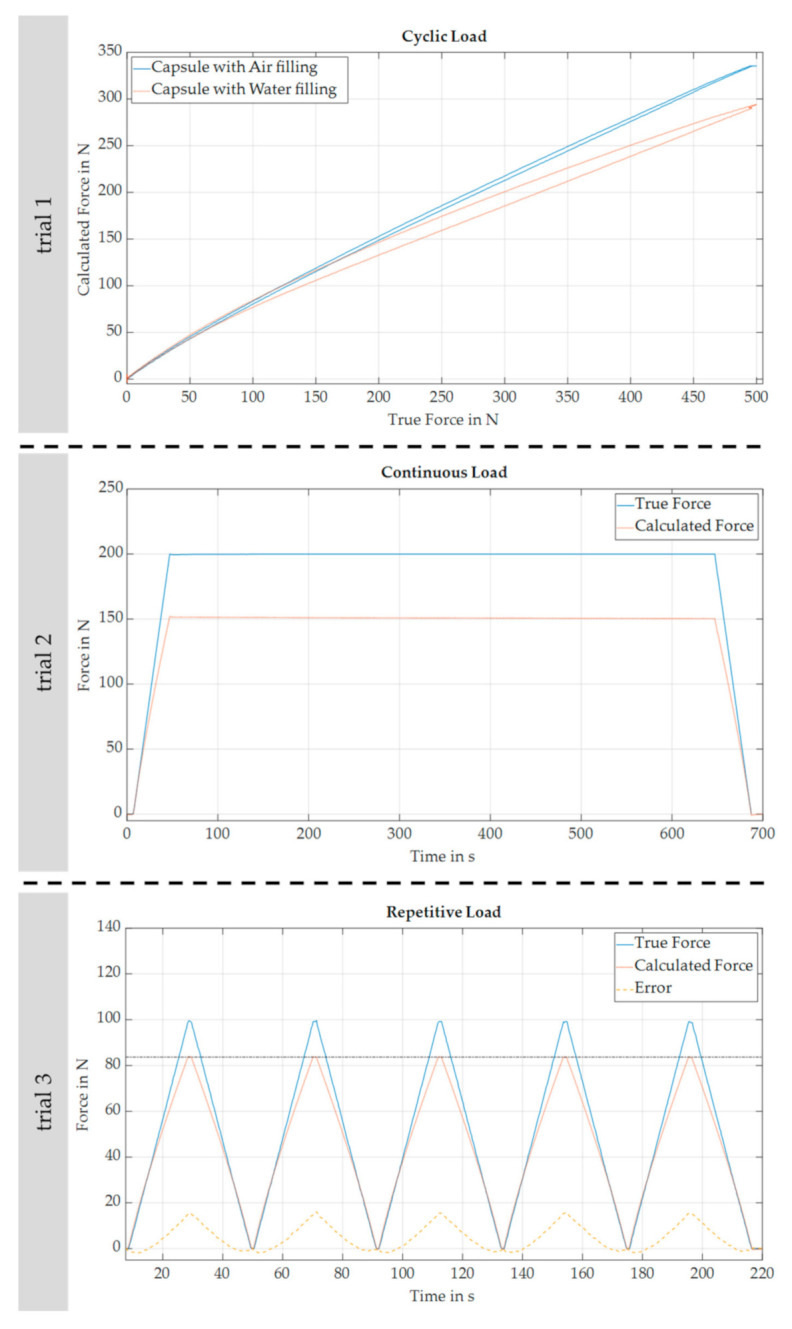
Measurement results in an isolated testing environment. The abscissa represents either the true force (see trial 1) or the elapsed time (see trial 2 and 3) in the respective trial. The ordinate always displays the calculated force of the sensor pad as well as for trial 2 und 3 the true force as well.

The developed sensor pad was clamped in the testing machine and both measurements’ data were stored and plotted in MATLAB R2018b. The measured force of the testing machine was assumed to be the true force and used as a reference. Based on the mentioned three-point-calibration for the sensor pad’s inner pressure in three different load stages, the respective force applied on the sensor pad was calculated (named as calculated force). Figure 5 shows the results for the different trials. The first and second trial are one cycle each, whereas the third trial has five repetitive cycles.

In principle, the use of an incompressible fluid such as water was recommended by Lee et al. [24] for the basic construction of the sensor pad. However, this hampers the measurement accuracy and ease of use in practice [29]. As illustrated in the results of the first trial, the replacement with the medium air improves the measurement accuracy of the sensor pad since the calculated force is closer to the true force for larger loads. An absolute measurement error of more than 5 newtons appears for the medium water at a threshold of 60 newtons (upwards) and 43 newtons (downwards) as well as for the medium air at a threshold of 52 newtons (upwards) and 38 newtons (downwards). However, the absolute measurement error is bigger for water after reaching the threshold of 110 newtons. Besides, the hysteresis of the sensor pad is less developed for the medium air since the calculated force for a true force of 250 newtons is 174.7 newtons (upward) and 159.3 newtons (downward) for the medium water or 185.8 newtons (upward) and 181.3 newtons (downward) for the medium air, respectively. Thus, the following trials and the real application on an exoskeletal interface were only conducted with silicon capsules filled with the medium air. In addition, it can be shown that the developed sensor pad unproblematically withstands a singular maximum load of 500 newtons. Here, no bursting of the silicon capsule with a resulting air lack was detected since the same silicon capsule could be used for the following trials with similar results compared to the measurements previous to the maximum load test.

As shown in the second trial, the sensor pad features a stable measurement behavior without drifts since the calculated force is almost constant during a constant true force of 200 newtons for 10 min. At the beginning, the calculated force is 151.5 newtons and 150.4 newtons after 10 min.

In the third trial, a linearly rising and declining true force was repeatedly applied to the sensor pad. It can be shown that the calculated force is almost the same in the different cycles. However, the absolute measurement error (see the dotted yellow line) is firstly negative and then nonlinearly increases with the load level. Finally, the maximum error is 15.7 newtons for a true force of 100 newtons.

### 3.2. Application on Exoskeletal Interface (Experiment 2)

Three sensor pads were embedded with a spatial array in the interface of the active exoskeleton “Lucy 2.0” [33] in order to assess their measurement accuracy in a realistic environment. Thus, the data of all sensor pads as well as of both additional, high-resolution measurement equipment were simultaneously recorded in LabVIEW, stored, and plotted in MATLAB R2018b.

**Figure 6:**
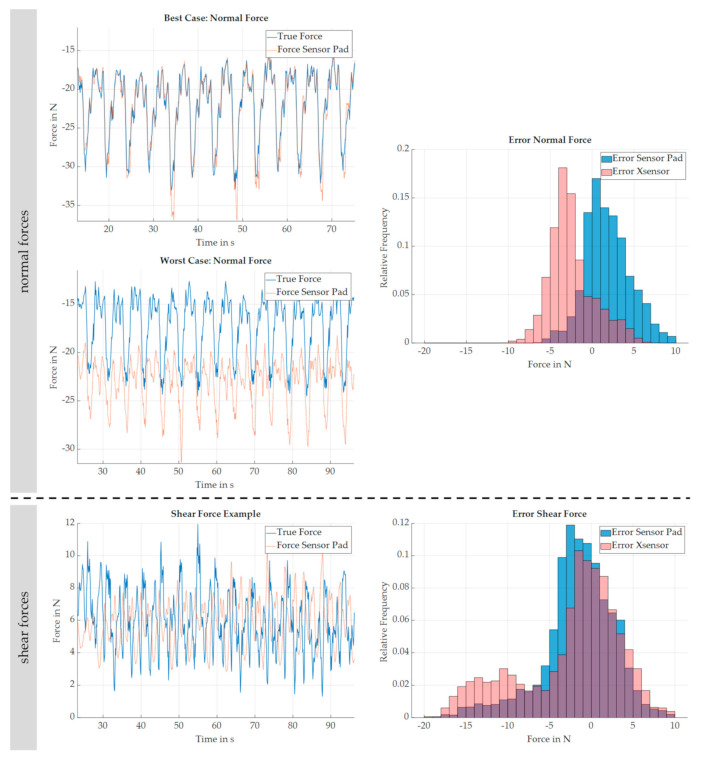
Comparison of measured forces in a real testing environment. Concerning the exemplary data plots (**left** side), the ordinate represents the true (blue, measured by force–torque sensor) or calculated forces (red, measured by sensor pad(s)). The abscissa displays the elapsed time for the trial. Concerning the charts for the measurement-error distribution (**right** side), the ordinate represents the probability of occurrence as a relative frequency. The abscissa displays the respective deviation of the pressure map (orange) and the sensor pad (blue) against the measurement of the force–torque sensor.

In principle, the analysis of measurement data focuses on the detection of normal interaction forces since the high-resolution pressure map from Xsensor is only capable of detecting forces in this direction. The high-resolution force–torque sensor of Schunk acts like a benchmark and is assumed to be the true force. Based on the spatial array, the sensory data of the sensor pads A and B as well as of their marked area on the pressure map are, respectively, compared to the measured force in F*_z_* of the force–torque sensor. This measured force is assumed to be the supportive force of the exoskeleton. Equally, the sensor pad C, its area on the pressure map, and the measured force in F*_x_* of the force–torque sensor are compared, which is assumed to be the shear force on the exoskeletal interface. Since the different measurement data are measured in different frequencies, the raw data of each equipment were linearly interpolated to match frequencies and automatically synchronized by shifting signals to the point of maximum cross correlation. Figure 6 illustrates the comparison of the respective force detections. The left side shows exemplarily chosen trials of different test persons. The measurement-error distribution on the right is derived by observing the respective total error as the difference between the true and the measured (Xsensor) or the calculated (sensor pad) force(s). As shown in the figure, the data of every test subject performing a minimum of ten motion repetitions are considered.

Firstly, the data for detecting normal forces on the exoskeletal interface are presented. It can be shown that the measurement accuracy depends on the test subject since the deviation is different between the two exemplarily shown measurement plots. This is also emphasized by the correlation matrix in Table 1 that shows the respective correlations for three exemplarily chosen test subjects for one trial. However, the chart for the measurement-error distribution considering the data of all test subjects shows that the probability of wide deviations is generally low, and most deviations are smaller than 5 newtons. Besides, the sensor pads principally underestimate and the pressure map overestimates the true force more likely. In addition, the sensor pad calculates the true force more accurately than the high-resolution pressure map, since the median is closer to the true force and the mean deviation of the sensor pad and the pressure map is 1.86 ± 2.70 and 2.14 ± 11.61 newtons, respectively.

Concerning the measurement of shear forces on the exoskeletal interface, the calculated force of the sensor pad (and the pressure map) does not generally correspond to the true force (see Table 1). Thus, it is less likely to correctly estimate the true force. A standard deviation of more than 10 newtons also appears frequently. The standard deviation considering the data of all test subjects for the sensor pad and the pressure map is 1.55 ± 4.32 and 2.39 ± 6.04 newtons, respectively.

## 4. Discussion

All in all, in our case, the presented sensor pad shows a prospectively promising competitive measurement performance compared to other high-resolution, expensive, and professional measurement equipment for human–machine interaction such as the multi-axis load cell or the pressure map. The main strength of the sensor pad in its presented form is its low-budget construction, its simplicity in form adaption to other possible application scenarios with adapted molds, its safety in use with external loads up to 500 newtons (or even beyond), and its accuracy up to 52 newtons as well as its general stability and repeatability in force detection. This seems to be beneficial according to other low-cost force sensors [26,27].

Besides, it can be constantly embedded in different human–machine interfaces. In this case, the sensor pad with its flexible air capsule can detect occurring interaction forces as well as bolster the interface and replace any additional foam [29]. Its skin-friendly silicon enables a longer direct skin contact. According to the exemplarily embedding in an exoskeletal interface, the current interface construction with three sensor pads arranged in a spatial array is capable of reliably detecting normal (supportive) forces on the interface (with sensor A and B), but shear forces might not be accurately measurable (with sensor C). The measurement accuracy generally seems to depend on the fit to the test subjects’ limbs, implying that the designed interface should be individually adjusted to test subjects in future studies. Besides, one test subject had outliers for the pressure map since the measurement data were much higher in reference to the other measurement data. This distorts the presented error histogram, and thus mean and standard deviation, for the pressure map, which should be less than the actual force. One possible reason might be kinkings of the sensor surface.

### 4.1. Potential for Embedding in Exoskeletons

In general, the embedding of force sensors in exoskeletal interfaces might enable the knowledge, analysis, interpretation, and assessment of occurring interaction forces in multiple directions. This might include statements concerning, e.g., the system’s level of support, dynamic behavior, fit, or comfort [29]. Besides, it offers the potential for (real-time) system adaptations—in particular for active exoskeletons and in combination with other sensory data such as inertial or electromyography sensors acting as a sensory network [22,35]. Possible initiatives are improved situation detections, adapted actuators or support curves, and changes in design, shape, orientation, or stiffness of the exoskeletal interfaces. The identification of appearing (task- or user-specific) patterns or thresholds might enable the application of (data-driven) machine-learning algorithms or artificial intelligence [36]. This should be outlined by the following example.

**Figure 7:**
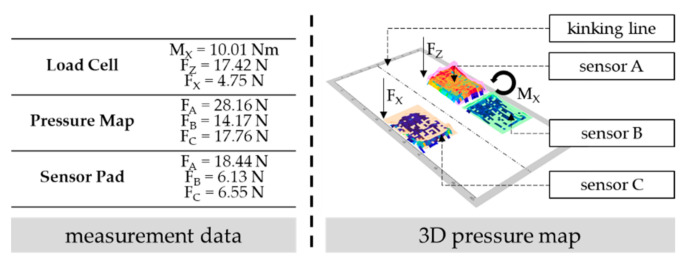
Comparison of the measured or calculated forces/torques at the same point of time of the respective measurement equipment (**left**) as well as a planar 3D-illustrated pressure map by the software from Xsensor (**right**).

Figure 7 on the left summarizes the data of the different measurement equipment at the same point of time, when one test subject is in a certain posture during the described arm motion in the second experiment. The respective exoskeletal support against gravity (namely, the normal force component) at this point of time might be derivable if F_Z_ (for load cell) or F_A_ together with F_B_ (for pressure map and sensor pad) are trigonometrically considered with the corresponding shoulder angle. Alternatively, the supportive moment on the shoulder can be calculated with the (measured or calculated) normal force on the interface and its lever arm to the shoulder joint [34]. The calculated support might be proven in a prospective study with a simultaneous detection of the shoulder angle, an analysis of the corresponding muscle activity [34], and a comparison with rated exoskeletal power outputs (see implemented force curves for active systems or material constants for, e.g., spring actuators of passive systems).

Besides, the illustration of pressure states on the surface of the pressure map (see Figure 7 on the right) shows an unequal force distribution with a focal point on sensor A. Thus, the exoskeletal interface does not fit well in this situation, since the human limb and exoskeletal interface axes are not parallel to each other, which leads to load peaks, might enable perceived discomfort, and requires system adjustments [25]. On the sensory side, the unequal distribution is detected by the occurring torque in M_X_ (see load cell) and the unequal force values of sensors A and B (see pressure map and sensor pad). The higher the torque or the disbalance of force values, the higher the load peaks on a certain body-surface area. In the future, certain thresholds that require system adjustments need to be defined based on investigations with forced disbalances and subjectively perceived discomfort of several test persons.

In addition, the course of occurring interaction forces in exoskeletal interfaces for certain tasks with varying motions and dynamics as well as for different system users should be prospectively investigated, since the respective courses vary in slope, leaps, and peaks [36]. Here, the analysis of patterns, key marks, and thresholds might enable system adaptations, (morphological) design selections, system evaluations, or derived system recommendations for certain user groups or application fields.

### 4.2. Ways for Improving the General Measurement Accuracy of the Sensor Pad

**Figure 8:**
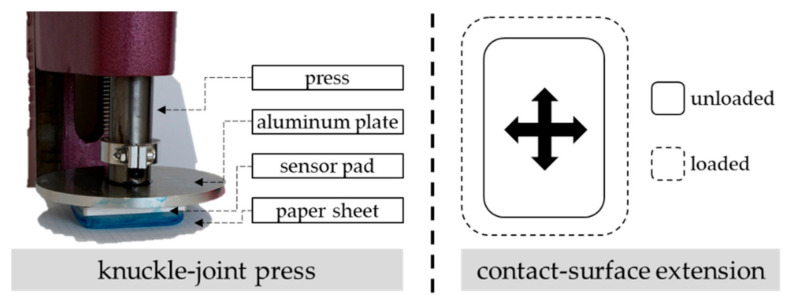
Knuckle-joint press with clamped sensor pad and inked silicon capsule on paper sheet (**left**). Schematic contact-surface extension of the sensor pad due to external load (**right**).

In general, the sensor pad in its current use shows a measurement weakness in both small- and high-measurement ranges. The second experiment reveals a negatively calculated force value for very small forces. As proven in the first experiment, the measurement error exceeds a value of ±5 newtons beyond an applied force of more than 52 newtons. Here, the error further increases with a rising measurable force, whereas the calculated force is always less than the true force. The reasons for this behavior mainly lie in the absolute term of the linear calibration [29] as well as the flexibility of the silicon capsule. This is proven in an additional experiment, where the sensor pad was inked with engineer’s blue and clamped into a knuckle-joint press with a maximum force of 17,000 newtons (see Figure 8). The press loaded the flexible silicone capsule with different normal forces ranging from 25.0 to 494.3 newtons so that the respective contact surface of the silicon capsule was marked blue on a white sheet of paper.

Table 2 presents the varying maximum contact surfaces for exemplarily applied loads on the silicon capsule.

All in all, several adjustments or initiatives on either a hard- or a software level (or both) might prospectively improve the measurement accuracy of the sensor pad, whereas a linear behavior of the applied piezoresistive pressure sensor MS5803-01BA is always assumed:

- Construction (hardware):
  One approach can be a redesign of the shape of the silicon capsule. This can imply the wall’s height or thickness, the form of the contact surface’s edge with an angular or chamfered edge, or the usage of a stiffer silicon with less material flexibility.

- Calibration (software):

An approach can be to expand the calibration range in the linear calibration with either more than three points or a broader load range beyond 100 to 500 g. Alternatively, the calibration can use certain offset algorithms (e.g., concerning the flexibility), alternative calibration types, or different calibrations for certain force ranges.

In addition, it is assumed that the measurement accuracy of the sensor pad in short and dynamic use is not dependent on the barometric pressure since the respective force calculation is based on the relative change against no load. However, a longer period of use, or static measurements, might require the consideration of changes in the barometric pressure (in particular during changing weather fronts). Here, the implementation of a barometric sensor for calculating an offset in calibration is recommended.

## Figures and Tables

**Table 1:** Correlation matrix with respective correlation coefficients for the detection of normal and shear forces, exemplarily applied on three different test subjects.

		Normal Forces	Shear Forces
Test Subject 1		**Force–Torque Sensor**	**Force–Torque Sensor**
	**Force–Torque Sensor**	1	1
	**Sensor Pad**	0.68	−0.34
	**Pressure Map**	0.69	−0.01
Test Subject 2		**Force–Torque Sensor**	**Force–Torque Sensor**
	**Force–Torque Sensor**	1	1
	**Sensor Pad**	0.84	0.19
	**Pressure Map**	0.68	0.21
Test Subject 3		**Force–Torque Sensor**	**Force–Torque Sensor**
	**Force–Torque Sensor**	1	1
	**Sensor Pad**	0.99	0.56
	**Pressure Map**	0.98	0.48

**Table 2:** Surface extension of the sensor pad due to external load.

Load (Newton)	Contact Surface (Square Centimeters)
25.01	5.44 × 4.15
49.52	5.67 × 4.36
102.97	5.96 × 4.63
171.61	6.20 × 4.85
494.26	6.63 × 5.27

## Data Availability

Not applicable.
